# Concurrent Metastatic Clear Cell Renal Cell Carcinoma and Chronic Lymphocytic Leukemia Within the Same Lymph Node: A Case Report and Review of Dual Malignancy Management

**DOI:** 10.7759/cureus.87448

**Published:** 2025-07-07

**Authors:** Paul F Hanona, Morgan Kiryakoza, Ping Zhang, George Howard

**Affiliations:** 1 Hematology Oncology, Corewell Health East, Royal Oak, USA; 2 Pathology, Corewell Health East, Royal Oak, USA; 3 Oncology, Corewell Health East, Royal Oak, USA

**Keywords:** chronic lymphocytic leukemia, cll, immunotherapy, pembrolizumab, rcc, renal cell carcinoma, urology

## Abstract

Dual malignancies involving clear cell renal cell carcinoma (ccRCC) and chronic lymphocytic leukemia (CLL) present a rare and challenging clinical scenario. This report describes a 75-year-old male with a history of monoclonal B-cell lymphocytosis (MBL) that progressed to CLL, later complicated by the development of ccRCC. Following neoadjuvant pembrolizumab, he underwent left nephrectomy with pathology confirming renal cell carcinoma (RCC). Subsequent imaging revealed a recurrent renal bed nodule, and a retroperitoneal lymph node biopsy demonstrated concurrent CLL and metastatic RCC. Our report aims to enhance recognition and management strategies for such complex dual malignancies.

## Introduction

Chronic lymphocytic leukemia (CLL) is seen in one out of every three new diagnoses of leukemia and accounts for nearly 1% of overall malignancies [1]. CLL is characterized by a proliferation of B cells that are functionally incompetent, an identical pathophysiology to small lymphocytic lymphoma (SLL). CLL is the term used when the abnormal B cells are present in the blood with an absolute lymphocyte count greater than 5,000/µL, whereas SLL is used when the lymph nodes are primarily affected by the aforementioned B cells with an absolute lymphocyte count less than 5,000/µL. CLL is typically asymptomatic at presentation; however, when symptomatic, it manifests as fatigue, recurrent infections, and painless lymphadenopathy. Flow cytometry is the most accurate diagnostic study, with CD5, CD19, CD20, and CD23-positive B cells. The hallmark on peripheral blood smear is a smudge cell, a phenomenon caused by the rupture of these fragile B cells during slide preparation. Treatment is usually reserved for symptomatic patients only [2,3].

Renal cell carcinoma (RCC) is the most common malignancy of the kidneys, representing nearly 90% of all cases of kidney cancer [4]. It originates from the proximal renal tubular epithelium, with smoking as the primary risk factor for development. In regard to genetic alterations, abnormalities in the short arm of chromosome 3 are usually the primary culprit. The most common symptom at presentation is hematuria, and an abdominal mass may be palpated on physical examination. The best initial imaging is a contrast-enhanced computerized tomography (CT) scan, and further histological confirmation is necessary for diagnosis. Treatment is typically surgical, with systemic therapy reserved for patients without surgical options [5].

Chatzikonstantinou et al. conducted an international, multicenter study that looked at just under 20,000 patients with CLL. They found that at least 21% of these patients had a secondary malignancy in addition to CLL. With such a prevalence, it is important to maintain a high clinical suspicion for secondary malignancies in CLL [6]. The co-occurrence of RCC and CLL is not commonly described; however, there appears to be some association between the two, with multiple case series published on the topic. Despite these multiple studies, an exact mechanism has not been elucidated. Some hypotheses suggest that secondary malignancy development may be due to treatments like chemotherapy, there may be a potential shared genetic mutation between the two, and the chronic inflammatory state that is CLL could also contribute to the development of RCC [7]. Given this potential association, further research is needed to better define the relationship.

## Case presentation

A 75‐year‐old male with a long-standing history of monoclonal B-cell lymphocytosis, first detected in 2016 through routine blood work demonstrating persistent lymphocytosis, presented for an oncologic evaluation regarding kidney cancer and CLL. His condition remained indolent until further workup four years later, when a chest CT, performed to follow up on a known lung nodule, revealed mild axillary adenopathy and borderline mediastinal nodes. An excisional biopsy of a right axillary lymph node the same year confirmed SLL/CLL with focal areas of increased Ki‐67 and cytogenetic abnormalities (trisomy 12 and 11q deletion). Despite these findings, the patient remained asymptomatic and was managed with observation.

**Figure 1:**
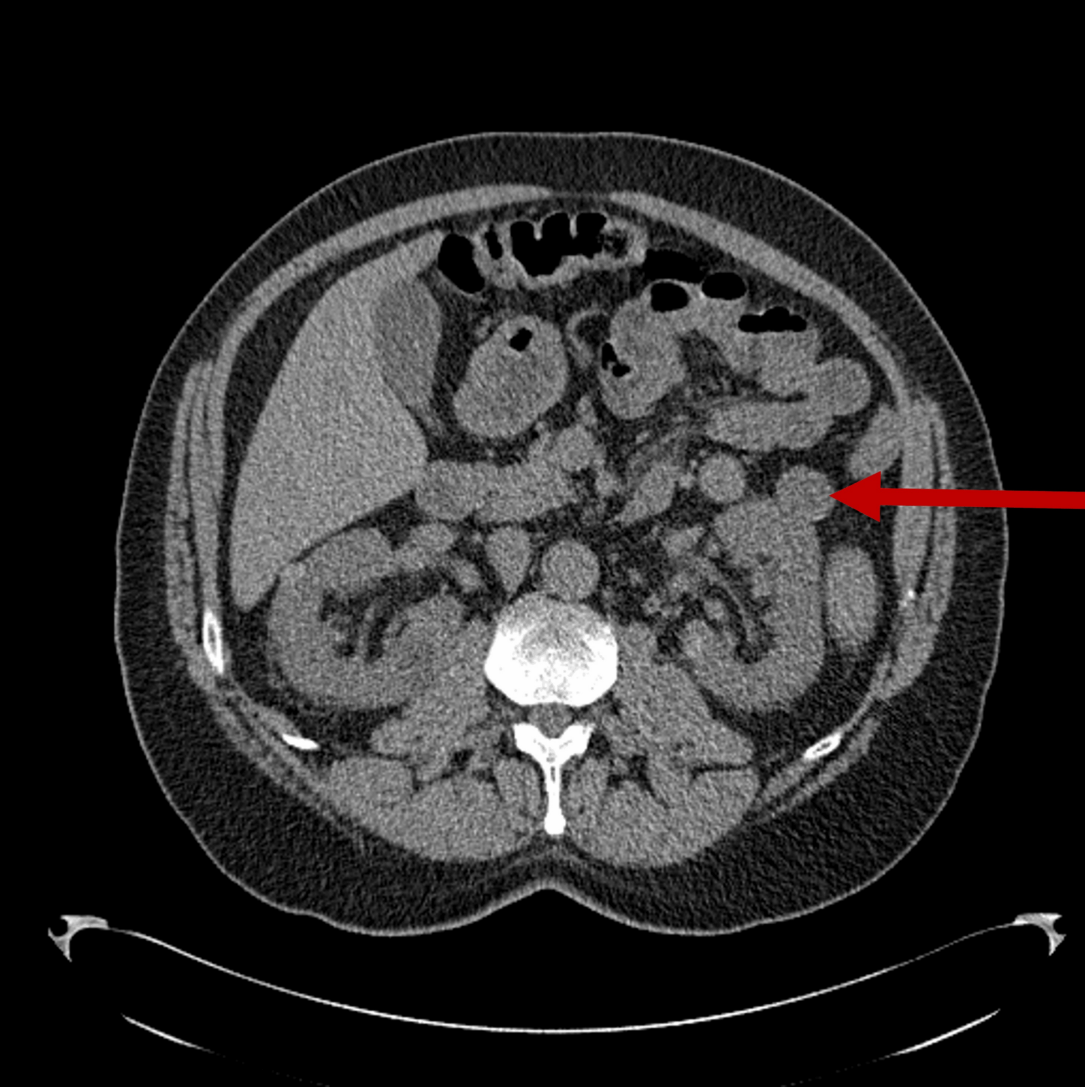
CT of the thorax in September 2020. A CT of the thorax done in September 2020 for lung cancer screening detected an initial renal mass on the left (red arrow).

**Figure 2:**
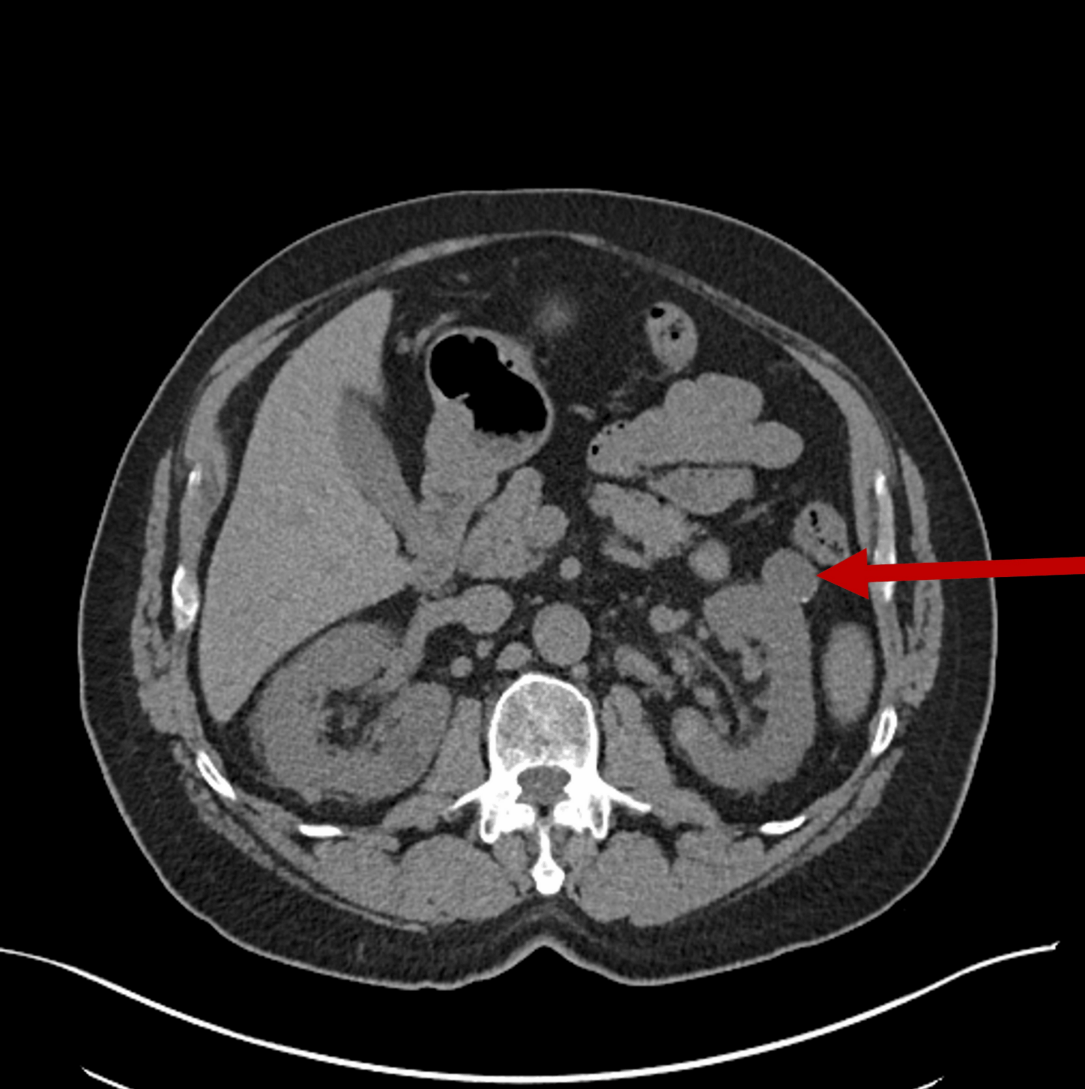
CT of the abdomen in February 2021. A CT of the abdomen with and without contrast in February 2021 further illustrates the left renal mass (red arrow).

Three years after his initial lymphocytosis, a screening lung cancer CT scan performed due to his significant smoking history identified a left renal mass (Figure 1). He subsequently underwent dedicated imaging, which further characterized this renal mass (Figure 2). Following multidisciplinary discussion, he received three months of neoadjuvant pembrolizumab prior to undergoing a left nephrectomy on October 30, 2022. Surgical pathology confirmed clear cell renal cell carcinoma (ccRCC), establishing his first diagnosis of a solid tumor malignancy. Postoperatively, his oncologic management focused on surveillance of both the nephrectomy bed and his known CLL.

On April 6, 2023, a CT scan demonstrated a 1.1 × 1.6 cm soft tissue nodule at the inferior aspect of the left nephrectomy bed, raising concern for RCC relapse. By April 23, 2023, treatment with a combination of pembrolizumab and axitinib was initiated. Due to treatment-related toxicities, including elevated liver enzymes and episodes of diarrhea, axitinib was temporarily held and later resumed at a reduced dose (3 mg twice daily), while pembrolizumab was continued at 20 mg IV every three weeks. Serial imaging revealed dynamic changes in the renal bed lesion: a CT scan on July 20, 2023, documented an increase in size to 2.1 × 1.5 cm, and a PET scan on August 1, 2023, measured the lesion at 2.3 × 1.4 cm with a maximum standardized uptake value of 3.79. Subsequent CT studies on October 31, 2023, and February 17, 2024, showed a reduction in the nodule’s dimensions to approximately 1.6 cm, with a PET scan on June 18, 2024, confirming further decrease and absence of tracer avidity.

**Figure 3:**
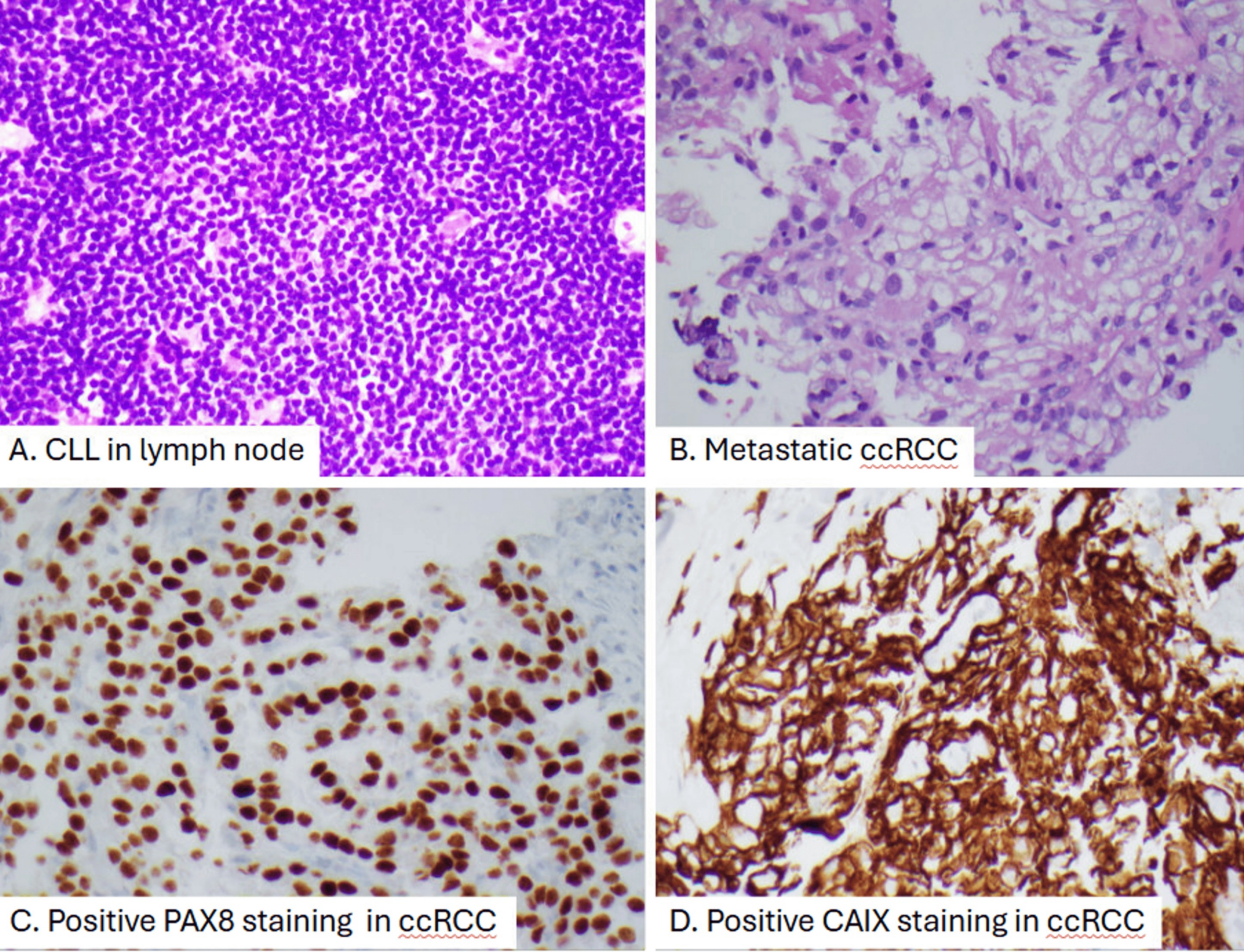
Pathological slides showing concurrent CLL and ccRCC. Panel A reveals monotonous small lymphocytes consistent with CLL. Panel B shows a carcinoma with clear cell cytoplasm and grade 3 nuclei on hematoxylin and eosin-stained sections, consistent with metastatic renal cell carcinoma. Panels C and D reveal positive nuclear PAX8 staining and cytoplasmic CAIX staining in tumor cells. CLL: chronic lymphocytic leukemia; ccRCC: clear cell renal cell carcinoma.

Given persistent generalized adenopathy on imaging, a retroperitoneal core biopsy was performed on September 19, 2024. Histopathologic evaluation of the specimen, which consisted of multiple tan-pink cylindrical cores, revealed dual pathology: the lymphoid tissue was consistent with his known CLL/SLL, while immunohistochemical studies demonstrated fragments of metastatic ccRCC (grade 2) within the same lymph node. Immunostains, including PAX-8 and CAIX, were positive, supporting the diagnosis of metastatic RCC, whereas markers typical for CLL/SLL were concurrently present (Figure 3).

On physical examination, the patient was well-nourished, in no acute distress, and exhibited stable vital signs. His laboratory values remained largely stable, with no significant cytopenias or biochemical abnormalities to suggest active disease progression. He continues to have painless lymphadenopathy. The patient continues to receive periodic pembrolizumab infusions, with ongoing surveillance of both the RCC relapse and CLL.

## Discussion

Managing concurrent ccRCC and CLL presents a challenging clinical scenario. This case presentation offers insights into the management of this rare concurrent presentation. There is already an established association between CLL and secondary primary malignancies. Falchi et al. show that patients with CLL have approximately a 1.2 to 2.2-fold increased risk of developing a second cancer compared to the general population [8]. Furthermore, RCC has a 1.5 to 2.5 times higher likelihood of occurring with CLL [9]. Our case highlights the progression of B-cell monoclonal gammopathy to CLL and then the concurrent development of ccRCC.

The pathophysiological link remains ambiguous. Solomon et al. proposed that immune dysregulation in CLL leads to an environment permissible for secondary malignancies [10]. Likewise, genetic dysregulation can lead to both CLL and ccRCC.

The management of dual malignancies presents unique challenges. Neoadjuvant pembrolizumab, followed by nephrectomy, is a standard approach for ccRCC. Ding et al. show that pembrolizumab may have activity in CLL, specifically with Richter transformation, suggesting a two-for-one approach [11]. When RCC recurred, our patient received pembrolizumab and axitinib as per standard RCC protocol. Rini et al. report improved progression-free survival with this combination when compared to sunitinib alone [12]. Treatment-related toxicities, as evidenced in our case, are a common barrier to the full completion of that regimen.

The dual pathology of CLL and ccRCC presenting within the same lymph node is a noteworthy finding. There may be some interaction in the tumor micro-environments of the two malignancies. Moreno et al. suggest that CLL can create a microenvironment suppressive enough to affect the growth of concurrent solid tumors [13]. CLL could have been the reason for this patient’s metastatic ccRCC presentation.

When considering surveillance, the changes in the renal bed highlight why multimodal imaging is required. This patient’s cancer responded originally to immunotherapy and targeted therapy, as evidenced by a reduction in metabolic activity. Ishimori et al. emphasized the importance of integrated PET/CT imaging in assessing treatment response in patients with dual malignancies [14].

Immunotherapy in patients with two malignancies that cross into the solid and hematological space is gathering attention. Pembrolizumab is a standard option for RCC, but the effects it has on CLL are unclear. Williams et al. report cases of CLL with higher infection rates following anti-CD-20 monoclonal antibody therapy [15]. Monitoring for disease progression and infections is paramount.

Duration of therapy and surveillance is also an evolving area in the world of CLL. Mato et al. proposed that individualized approaches be based on the individual tumor types and the particular condition of the patient [16]. For our patient, continuing pembrolizumab with interval imaging and laboratory testing represents an approach within the standard of care. This case also highlights the value of multidisciplinary collaboration. Stephenson et al. underscored that such collaborative approaches lead to improved outcomes in patients with complex oncologic presentations [17].

## Conclusions

In conclusion, our case demonstrates the successful management of concurrent CLL and metastatic ccRCC despite significant therapeutic challenges. Favorable outcomes can be achieved with targeted and immunotherapeutic approaches, especially when combined with proper monitoring. More research is needed into the biological interactions between coexisting solid and hematological malignancies.
